# Perspectives of Emergency Clinicians About Medical Errors Resulting in Patient Harm or Malpractice Litigation

**DOI:** 10.1001/jamanetworkopen.2022.41461

**Published:** 2022-11-10

**Authors:** Daniel Ostrovsky, Victor Novack, Peter B. Smulowitz, Ryan C. Burke, Bruce E. Landon, Linda M. Isbell

**Affiliations:** 1 Clinical Research Center, Soroka University Medical Center, Ben-Gurion University of the Negev, Be'er Sheva, Israel; 2 Department of Emergency Medicine, University of Massachusetts Medical School, Worcester; 3 Department of Emergency Medicine, Beth Israel Deaconess Medical Center, Boston, Massachusetts; 4 Department of Health Care Policy, Harvard Medical School, Boston, Massachusetts; 5 Division of General Internal, Medicine, Beth Israel Deaconess Medical Center, Boston, Massachusetts; 6 Department of Psychological and Brain Sciences, University of Massachusetts, Amherst

## Abstract

This cross-sectional study analyzes responses to a survey about medical error outcomes completed by emergency department attending physicians and advanced practice clinicians.

## Introduction

Malpractice litigation is a major concern when medical errors result in adverse patient outcomes. Fear of malpractice has been associated with excessive health care use through defensive medicine, whereby additional testing or referrals are made to protect physicians from malpractice accusations.^1^ Although fear of lawsuits and its implications for decision-making have been well studied,^2^ few investigators have examined clinicians' concern regarding causing patient harm, which might be a stronger impetus for excessive testing. This cross-sectional study investigated emergency department (ED) clinicians' concerns about medical errors resulting in either patient harm or malpractice litigation.

## Methods

We conducted an online survey targeting all ED attending physicians and advanced practice clinicians (APCs) in acute care hospitals across Massachusetts from January to September 2020. Detailed methods are described elsewhere.^3^ The Harvard Medical School Institutional Review Board approved this study. Respondents provided written informed consent. We followed the STROBE reporting guideline.

Respondents used a Likert scale of 1 (strongly disagree) to 6 (strongly agree) to indicate degree of agreement with 2 statements: “In my day-to-day practice, I am fearful of making a mistake which results in [1] harm to the patient” (fear of harm) and “[2] being sued” (fear of suit). Respondents reported their age, race and ethnicity, number of shifts per month, percentage of night shifts, years of experience, and salary type. Two-sided *P* < .05 indicated significance. R, version 4.1.2 (R Core Team), was used for analyses.

## Results

Respondents included 1222 clinicians (mean [SD] age, 43.3 [10.6] years; 608 men [54.2%], 506 women [45.1%], 8 other gender [0.7%]). Response rates were similar for physicians (77.2%) and APCs (75.8%). The Table shows respondent characteristics.

**Table.** Respondent Characteristics

Characteristic	Respondents, No. (%) (n = 1122)
Age, mean (SD), y	43.3 (10.6)
Sex	
Male	608 (54.2)
Female	506 (45.1)
Other^a^	8 (0.7)
Race and ethnicity^b^	
Asian	103 (9.2)
Black	23 (2.0)
White	959 (85.5)
Other^a^	37 (3.3)
Years of experience, median (IQR)	10.00 (5.00-19.00)
Clinician subtype	
MD or DO	782 (69.7)
APC	340 (30.3)
No. of shifts per month, median (IQR)	12.00 (10.00-15.00)
Percentage of night shifts, median (IQR)	10.00 (2.00-25.00)
Salary type	
Salary	370 (33.0)
Salary + bonus	607 (54.1)
Productivity-based	94 (8.4)
Other^a^	50 (4.5)
Fear-of-harm score, mean (SD)^c^	4.40 (1.32)
Fear-of-suit score, mean (SD)^d^	3.40 (1.41)

Abbreviations: APC, advanced practice clinician; DO, doctor of osteopathic medicine; MD, doctor of medicine.

^a^ Respondents selected *other* as an answer in this category.

^b^ Race and ethnicity were self-identified.

^c^ Fear of harm refers to the fear of making a mistake that results in harm to a patient.

^d^ Fear of suit refers to the fear of making a mistake that results in a malpractice suit.

Mean score was greater for fear of harm than fear of suit (4.40 vs 3.40; *P* < .001). Mean fear-of-harm score was similar regardless of survey completion before or after the COVID-19 pandemic started (4.42 vs 4.39; *P* = .70). No difference was found in mean fear-of-suit score before vs after the pandemic started, (3.41 vs 3.38; *P* = .70). Modal responses were *moderately agree* to fear-of-harm (31.1% [n = 349]) and *slightly agree* to fear-of-suit (29.1% [n = 327]) questions. Fear-of-harm scores were higher than fear-of-suit scores, regardless of clinician subtype, experience, or sex (Figure), and positively correlated (Pearson *r* = 0.53; *P* < .001).

**Figure.**
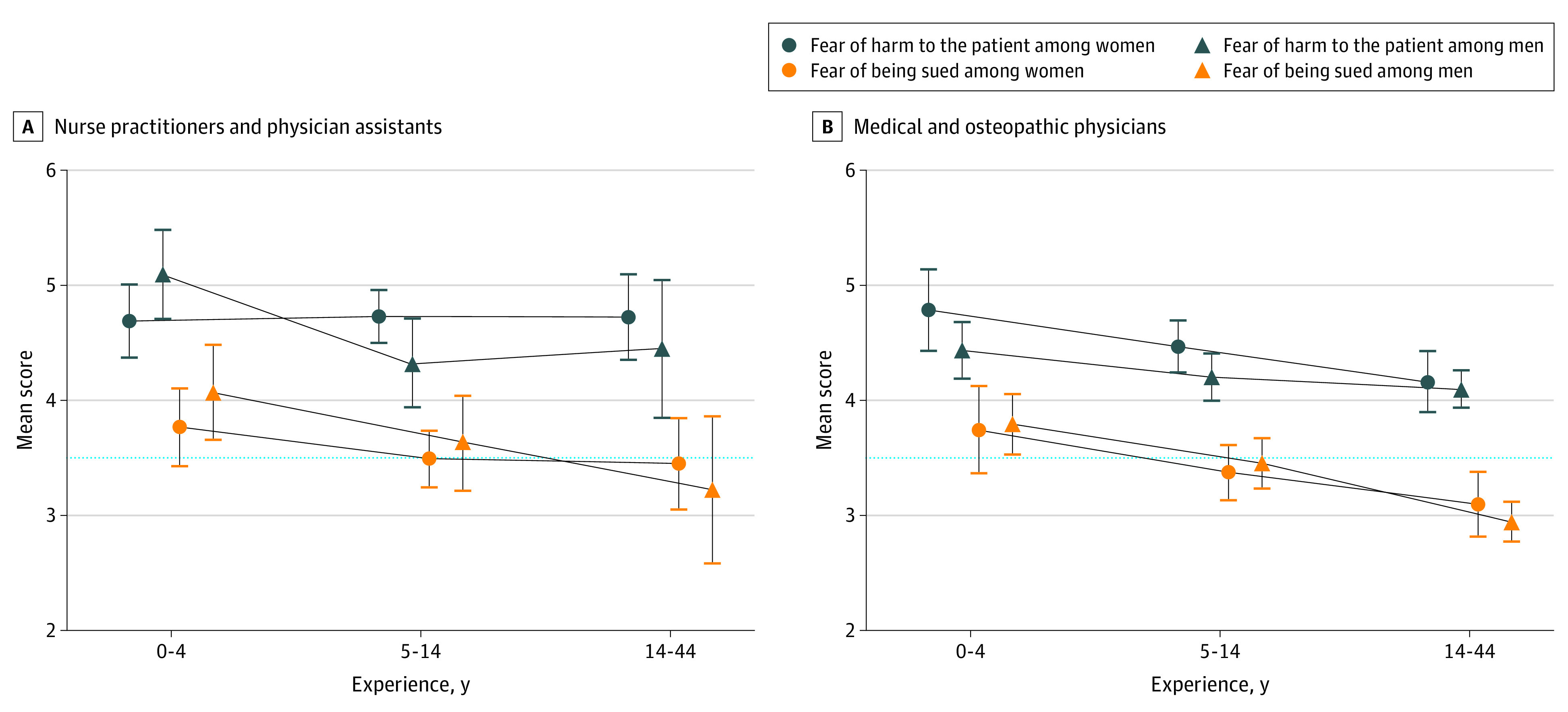
Crude Fear-of-Harm and Fear-of-Suit Mean Scores as a Function of Experience, Sex, and Clinician Subtype Error bars represent 95% CI. Dotted lines represent the border between agreeing and disagreeing responses.

In multivariable linear regressions, adjusted fear-of-harm score differed between respondents with 5 to 14 years of experience (regression coefficient [β] = –0.25; *P* = .009) and 15 to 44 years of experience (β = –0.38; *P* < .001) vs respondents with 0 to 4 years of experience. Differences emerged for physicians vs APCs (β = –0.30; *P* < .001) and male vs female respondents (β = –0.17, *P* = .03). Fear-of-suit score differed between respondents with 5 to 14 years of experience (β = –0.35; *P* < .001) and 15 to 44 years of experience (β = –0.72; *P* < .001) vs those with 0 to 4 years of experience and for physician vs APCs (β = –0.18; *P* = .053). Statistical differences by sex were not detected.

## Discussion

The findings of this study suggest that, although clinicians feared legal action, they feared harming patients to a greater degree regardless of clinician subtypes, experience, or sex; these findings are relevant to defensive medicine. Some studies implied that fear of lawsuit was the primary factor in excessive testing by physicians.^4^ Clinicians were also concerned about preventing patient harm. Although the study did not delineate the association between this concern and potential overuse of testing, it suggested that fear of harm should be considered with, and may be more consequential than, fear of suit in medical decision-making.

Study limitations included the sample of ED clinicians from across Massachusetts, which may limit generalizability. Moreover, although we achieved high response rates, we could not assess whether respondents differed from nonrespondents. Additional research is needed to elucidate the role of fear of harm, compared with fear of suit, in clinician decision-making.
